# Consistency of mouse models with human intracerebral hemorrhage: core targets and non-coding RNA regulatory axis

**DOI:** 10.18632/aging.205473

**Published:** 2024-01-24

**Authors:** Sinan Jin, Jincheng Meng, Chong Zhang, Jiping Qi, He Wu

**Affiliations:** 1 Department of Pathology, First Clinical Hospital, Harbin Medical University, Harbin 150001, China

**Keywords:** intracerebral hemorrhage, translational medicine, gene regulatory network, biomarkers, signaling pathway

## Abstract

Intracerebral hemorrhage (ICH) has a high mortality and disability rate. Numerous basic studies on pathogenesis and therapeutics have been performed in mice. However, the consistency of the experimental mouse model and the human ICH patient remains unclear. This has slowed progress in translational medicine. Furthermore, effective therapeutic targets and reliable regulatory networks for ICH are needed. Therefore, we determined the differentially expressed (DE) messenger RNAs (mRNAs), microRNAs (miRNAs) and circular RNAs (circRNAs) before and after murine ICH and analyzed their regulatory relationships. Subsequently, data on mRNAs from human peripheral blood after ICH were obtained from the Gene Expression Omnibus database. The DE mRNAs after human ICH were compared with those of the mouse. Finally, we obtained seven genes with translational medicine research value and verified them in mice. Then the regulatory network of these genes was analyzed in humans. Similarly, species homologies of these regulatory pathways were identified. In conclusion, we found that the mouse ICH model mimics the human disease mainly in terms of chemokines and inflammatory factors. This has important implications for future research into the mechanisms of ICH injury and repair.

## INTRODUCTION

Intracerebral hemorrhage (ICH) has high morbidity and mortality. 58% of ICH patients die within one year, and two-thirds of survivors become moderately or severely disabled [1, 2]. Animal research has enabled great progress in uncovering the mechanisms of ICH [3], and studies have shown that inflammatory factors and their signaling pathways play an important role in injury after ICH [4, 5]. Chemokines are also key regulators of the neuronal damage [6–8].

Competitive endogenous RNAs (ceRNAs) often regulate gene expression levels in a ceRNA-microRNA (miRNA)-messenger RNA (mRNA) axis and are thus involved in a wide range of regulatory mechanisms for biological processes [9]. Numerous studies have identified the involvement of ceRNAs in the regulatory mechanisms of neurological injury after ICH in mice [10]. They have been shown to be closely associated with the inflammatory response after ICH. However, because they are composed of shorter sequences, the sheer number of them makes their translational medical study more difficult [11]. In addition, there is a need to understand how animal experimental models can mimic human ICH disease. This will be very useful in guiding future mechanism research.

To address these issues, we obtained differentially expressed (DE) mRNAs, miRNAs and circular RNAs (circRNAs) before and after ICH in mice to obtain the complete differentially expressed ceRNA regulatory network. And the DE mRNAs were combined with the analysis of transcriptome data obtained from human peripheral blood after ICH. Here, we propose mRNA targets with translational medicine implications for future studies of ICH injury mechanisms in mice. We also provide several circRNA-miRNA-mRNA networks for these targets, all of which are significantly differentially expressed after ICH in mice. Identifying and reporting these networks will help to more effectively study secondary injury mechanisms after ICH.

## RESULTS

### Changes in gene expression profile in the mouse brain after ICH

**Figure 1:**
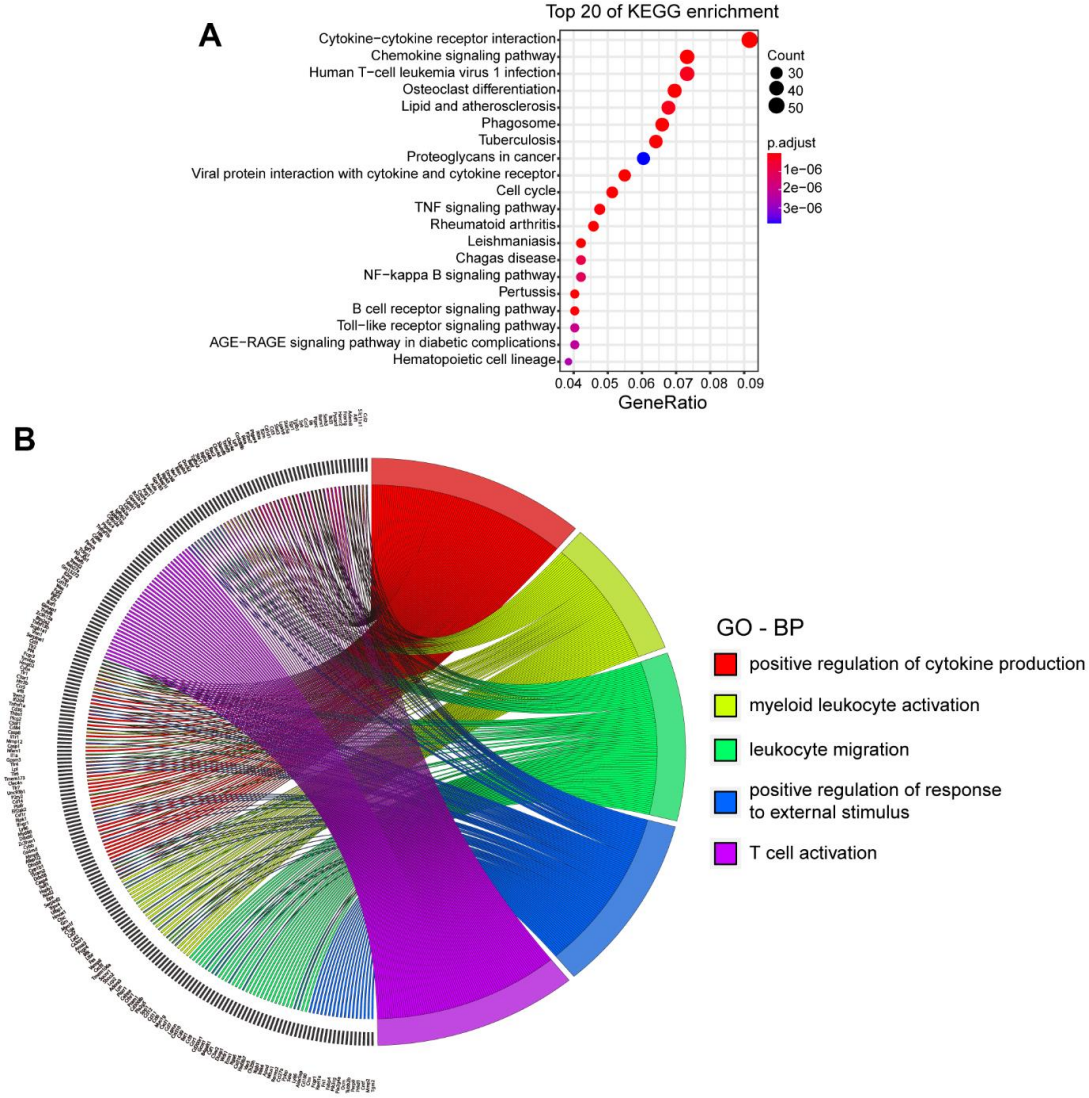
**KEGG and GO analysis of DEGs in mice with ICH.** (**A**) Top 20 Kyoto Encyclopedia of Genes and Genomes (KEGG) of differentially expressed genes (DEGs). (p.adj ≤ 3.86×10^-6^). (**B**) Top 5 biological processes (BPs) of Gene ontology (GO) and DEGs involved. (p.adj ≤1.28×10^-24^). (ICH 3 d vs. Con, n=3).

Three days after collagenase-induced ICH, the brains of the mice were harvested. Gene expression levels were determined and compared to healthy mice. A total of 39,483 genes were identified. The results showed an increase in the expression of 13,434 genes after ICH and a significant increase in 1093 genes. Of the 18,486 genes with decreased expression, 120 were significantly downregulated (|log2FC|>1 and p.adj<0.05, Supplementary Figure 1A). The top 20 Kyoto Encyclopedia of Genes and Genomes (KEGG) enriched pathways are shown in Figure 1A. The classical pathways were the AGE-RAGE pathway, the NF-κB pathway, the TLR pathway and the TNF pathway. Table 1 lists the differentially expressed genes (DEGs) involved in more than two pathways simultaneously.

**Table 1:** DEGs participate in four classical signaling pathways after ICH in mice.

	**AGE-RAGE signaling pathway in diabetic complications**	**NF-kappa B signaling pathway**	**Toll-like receptor signaling pathway**	**TNF signaling pathway**	**Log2FC**	**Adjusted p-value**
**Nfkb1**	•	•	•	•	1.03	0.016423
**Il6**	•		•	•	2.99	1.05E-06
**Icam1**	•	•		•	1.61	0.014022
**Ripk1**		•	•	•	1.73	3.63E-05
**Ccl12**	•			•	4.18	7.35E-28
**Ccl2**	•			•	5.63	8.61E-53
**Sele**	•			•	1.68	0.036437
**Plcg2**	•	•			1.96	5.70E-10
**Ly96**		•	•		1.46	0.000256
**Myd88**		•	•		1.29	0.000363
**Tlr4**		•	•		2.04	8.61E-07
**Cd14**		•	•		1.62	1.03E-05
**Ticam2**		•	•		2.06	0.003196
**Irak4**		•	•		1.64	0.001116
**Cxcl1**		•		•	3.76	8.55E-11
**Tnfrsf1a**		•		•	1.90	5.37E-12
**Casp8**			•	•	2.21	8.72E-08
**Fos**			•	•	1.45	0.044935
**Cxcl10**			•	•	3.03	4.26E-09
**Ccl5**			•	•	1.46	0.000563
**Map3k8**			•	•	1.15	0.014022

• indicates that the gene participates in the corresponding pathway.

The DEGs were analyzed by Gene Ontology (GO). The top five biological processes (BPs) and the genes involved are shown in Figure 1B. Up to 93 genes were involved in cytokine production, which was the top-ranked BP. Several well-known genes in this group were *Pf4*, *Tlr* family genes, *Hmgb2*, *Il6* and *Cd36*. We also examined the top five cellular components (CCs) and molecular functions (MFs) of all DEGs (Supplementary Figure 1B, 1C). The results showed that these genes tended to be expressed in the chromosomes and extracellular matrix. The top five MFs in which the genes were involved were immune receptor activity, integrin binding, cytokine binding, chemokine activity and toll-like receptor (TLR) binding.

### Changes in miRNA and circRNA expression levels in mouse brain after ICH

**Figure 2:**
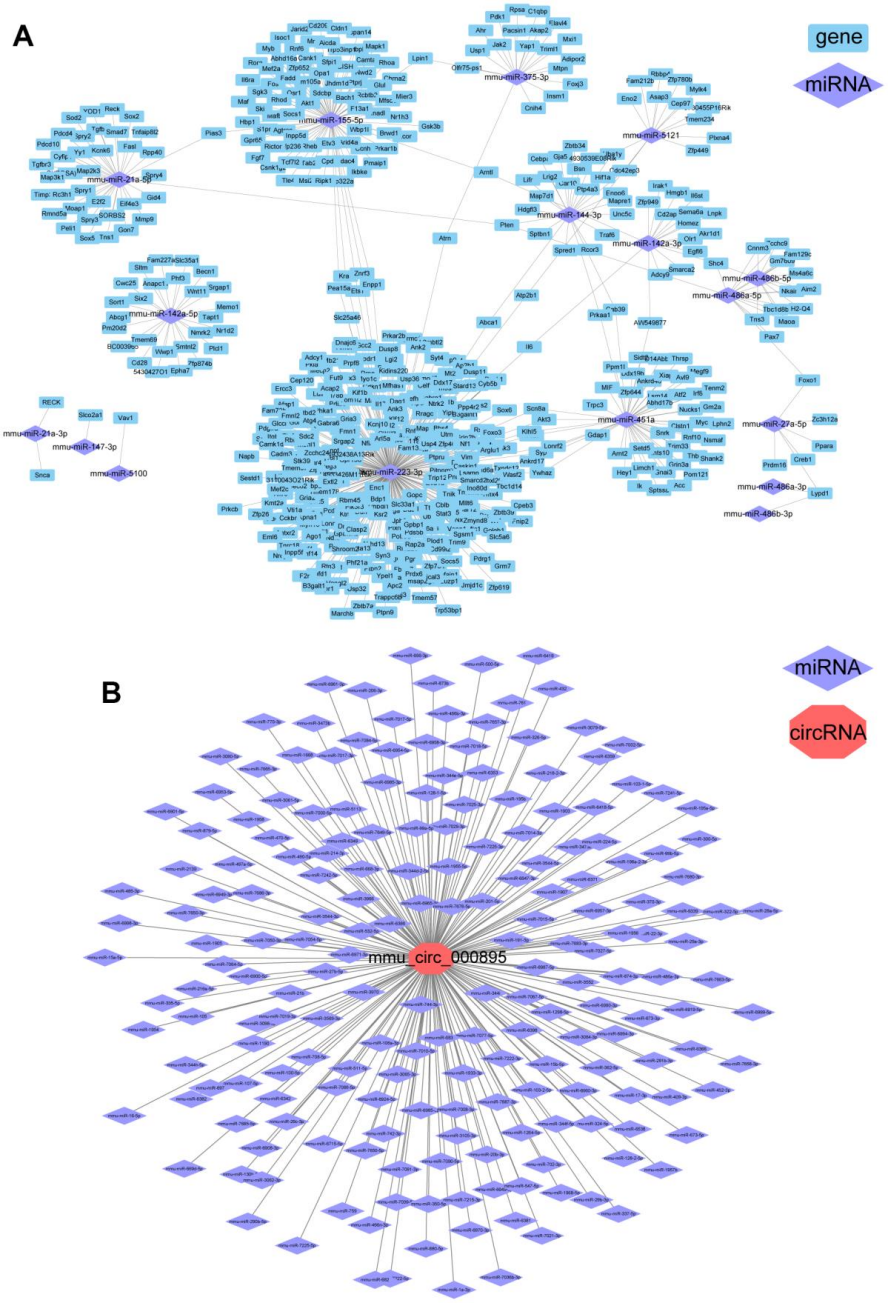
**Differential regulatory networks following ICH in mice.** (**A**) Regulatory network of differentially expressed (DE) miRNAs and DEGs in ICH mice. (**B**) miRNAs that may be targets of circRNA-000895.

A total of 1170 known miRNAs were identified in mouse brain. After data normalization, 22 miRNAs showed significant changes in the ICH 3 d group compared to the control group (|log2FC|>1 and p.adj<0.05, Supplementary Figure 2A). Among these, the expression of 21 miRNAs was upregulated and only mmu-miR-375-5p was significantly downregulated (log2FC=-1.7, p.adj = 1.25 × 10^-6^). A regulatory network of differentially expressed miRNA (miR)-gene was constructed (Figure 2A). It’s worth noting that up to 311 genes were identified as the target of mmu-miR-223-3p (log2FC=2.1, p.adj=2.54×10^-16^). The mmu-miR-223-3p, mmu-miR-451a and mmu-miR-155-5p were upregulated after ICH, and their important role in regulating gene expression levels has been demonstrated in the cerebrospinal fluid of patients with subarachnoid hemorrhage [12].

Next, we found 100 circRNAs with significant changes in expression levels in the ICH 3 d group. Among them, 71 were upregulated and 29 were downregulated compared to the control (Supplementary Figure 2B). Since circRNAs can inhibit the function of miRNAs by binding to them, predicting their binding sites is important to provide a theoretical basis for future research. Using miRanda and psRobot to predict binding sites, we identified one notable circRNA, mmu-circRNA-000895, which can bind to 199 miRNAs (Figure 2B) and was upregulated at ICH 3 d (log2FC=1.49, p.adj=4.6×10^-4^).

In addition, we summarize the DEGs and the ceRNA networks that can potentially be regulated by circRNA-000895: miR-214-3p-*Ccl9*, miR-761-*Ccl9*, miR-362-5p-*Itgb1*, miR-15a-5p-*Nfatc1*, and miR-511-5p-*Tnfrsf1a*. Furthermore, circRNA-000895 can regulate the expression of *Lypd1* by binding to miR-486a-3p or miR-486b-3p. In the transcriptome gene screening, the expression of Lypd1 decreased after ICH. Although its p-value was 0.02 and the false discovery rate was 0.13, it was not classified as a gene with significant changes after ICH because its FC was only 1.8. It can be speculated that circRNA-000895 may regulate multiple genes after ICH in a ceRNA-dependent manner.

### DEGs in human peripheral blood 72 h and 24 h after ICH

We searched the GEO database for a 0-24 h and 72-96 h human peripheral blood transcriptome and re-analyzed the raw data using bioinformatics (GEO accession number: GSE 125512). Data were obtained from eleven patients (four African Americans and seven white Americans) with a mean age of 57.5 years. Blood samples were taken 24 and 72 hours after the onset of ICH and transcriptome sequencing was performed. A total of 16,640 genes were identified in human peripheral blood. As all data are from post-onset blood, we have relaxed the fold change accordingly [13]. For genes with p.adj<0.05, log2FC≤-0.5 indicated that the genes were downregulated in ICH 72 h, and log2FC≥0.5 indicated that the genes were upregulated. Using these criteria, 456 genes were significantly upregulated and 183 were significantly downregulated in ICH 72 h (Supplementary Figure 3A).

**Figure 3:**
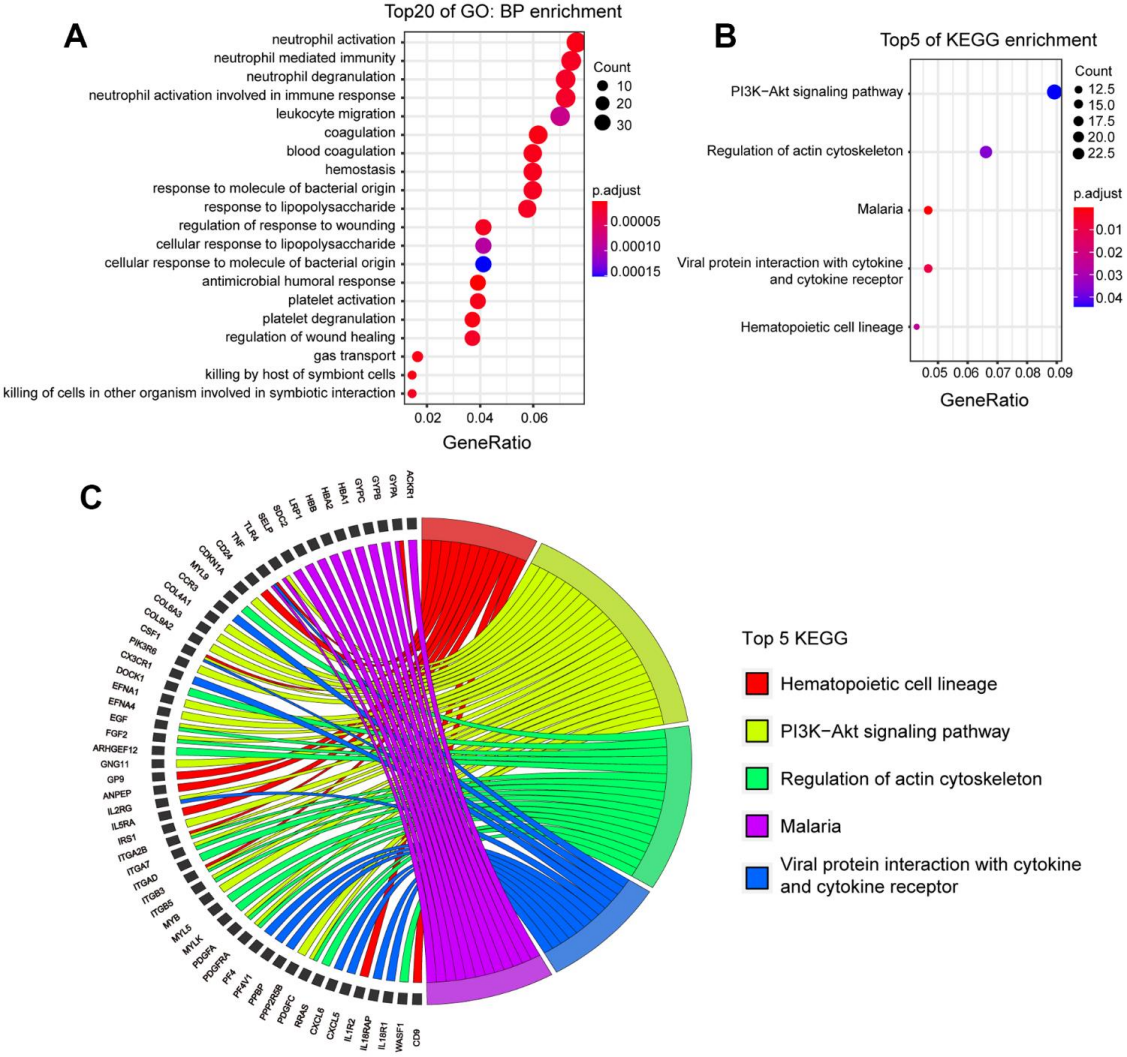
**KEGG and GO analysis of DEGs in human patients with ICH.** (**A**) Top 20 BPs enrichment of GO of DEGs. (p.adj ≤ 7.0×10^-6^). (**B**) Top 5 of KEGG enrichment of DEGs (p.adj ≤0.04). (**C**) The DEGs involved in top 5 KEGG. (ICH 72h vs. ICH 24h, n=11).

GO analysis was also performed on these DEGs. These DEGs were involved in 171 biological processes (Figure 3A). The first four BPs were related to neutrophil activity and these BPs involved the same 35 DEGs; These genes are expressed in the bone marrow under physiological conditions and were significantly up-regulated 72 h after ICH, indicating that ICH injury triggered a systemic inflammatory response on the third day after the onset of the disease. The results of the analysis of the top 20 CCs and MFs are shown in Supplementary Figure 3B, 3C.

**Figure 4:**
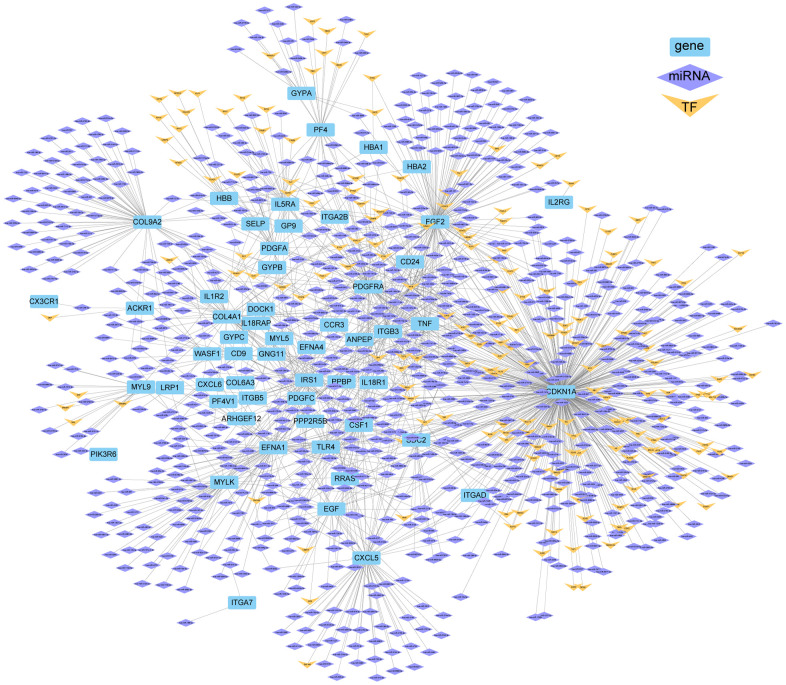
Network of TF/miRNAs and DEGs (top five KEGG pathways) in ICH patients.

KEGG analysis was then performed. Among the top five KEGG enrichment pathways in human peripheral blood DEGs (Figure 3B, 3C), four of them also appeared in the KEGG enrichment pathways of mouse DEGs: ‘PI3K-Akt signaling pathway’, ‘Malaria’, ‘Viral protein interaction with cytokine and cytokine receptor’, and ‘Hematopoietic cell lineage’. To further understand the regulation mechanism of these genes, networks of the top five KEGG DEGs were constructed (Figure 4).

### Seven genes significantly differentially expressed after human/mouse ICH

**Figure 5:**
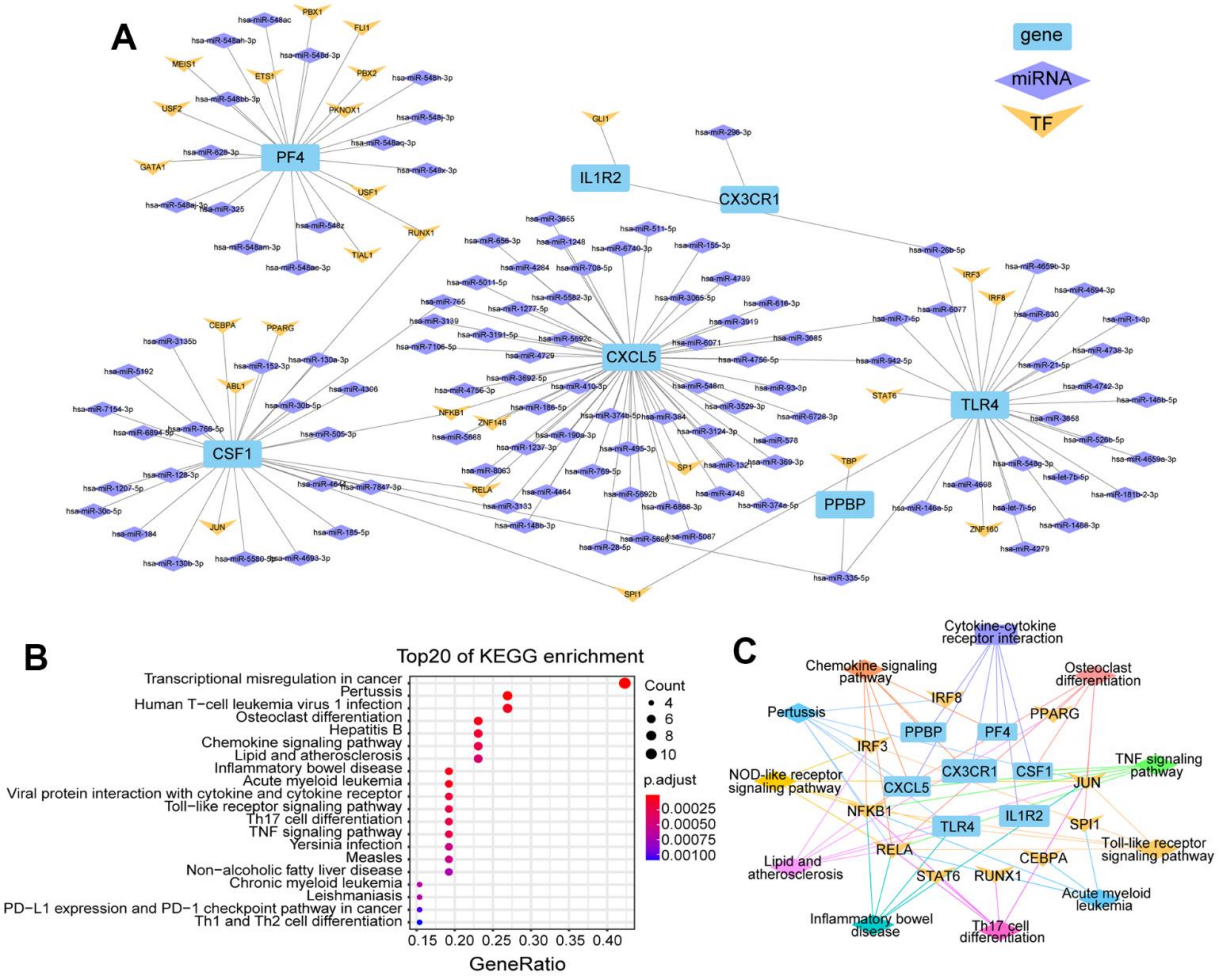
**Regulate network and enrichment analysis for the seven DEGs of human/mouse ICH.** (**A**) Genes differentially expressed in both human peripheral blood and mouse brain after ICH and their regulated network, including miRNAs and TFs. (**B**) The top 20 KEGG enrichment pathways involved with these genes and their TFs (p.adj ≤ 0.001). (**C**) The functional networks of these genes and TFs.

After analyzing the human and mouse ICH transcriptome data, the DEGs of the two species were integrated and seven identical genes were identified. These genes are: *Tlr4*, *Il1r2*, *Csf1*, *Cx3cr1*, *Cxcl5*, *Pf4* and *Ppbp* were all upregulated in mouse brain in ICH 3 d. However, in human peripheral blood, the expression levels of *TLR4* and *IL1R2* were higher at 24 h than at 72 h after ICH. The regulatory networks of these genes were then constructed from three aspects: TFs and miRNAs (Figure 5A). According to the predictions, 56 miRNAs target *CXCL5*, of which hsa-miR-765 and hsa-miR-148b-3p simultaneously target *CSF1*, and hsa-miR-7-5p and hsa-miR-942-5p target *TLR4*. In addition, hsa-miR-26b-5p targets *TLR4* and *IL1R2*, and hsa-miR-335-5p targets *CSF1*, *PPBP* and *TLR4*. In addition, NFKB1 and RUNX1 are both TFs of *CSF1* and *PF4*.

Next, KEGG enrichment analysis was performed for the seven genes and their TFs, and the data were visualized for the top 20 pathways (Figure 5B). A functional network of the seven genes and their TFs was also constructed (Figure 5C). NFKB1 and RELA, the TFs of *CSF1* and *CXCL5*, were involved in all pathways shown in Figure 5C, except cytokine receptor interaction. In addition, miR-30b/c-5p also have binding sites on *CSF1*. *CSF1* and *TLR4* share the same TF named SPL1. *CXCL5* and *CSF1* can be transcribed by the TFs NFKB1 and RELA and both have targeted binding sites with hsa-miR-148b-3p; this miRNA is also homologous in human and mouse. We also found that hsa-miR-335-5p targets *PPBP, CSF1* and *TLR4*. miR-335-5p is highly homologous in humans and mice. Notably, mmu-miR-335-5p has potential binding sites with mmu-circRNA-000895. It should be noted that the presence of hsa-SEMA3F_0001 has been detected in human urine exosomes (ID no. exo_circ_52661 in exoRBase v2). Therefore, the circRNA-000895-miR-335-5p-*PPBP/CSF1/TLR4* signaling pathways are highly relevant for translational medicine in the context of ICH.

**Figure 6:**
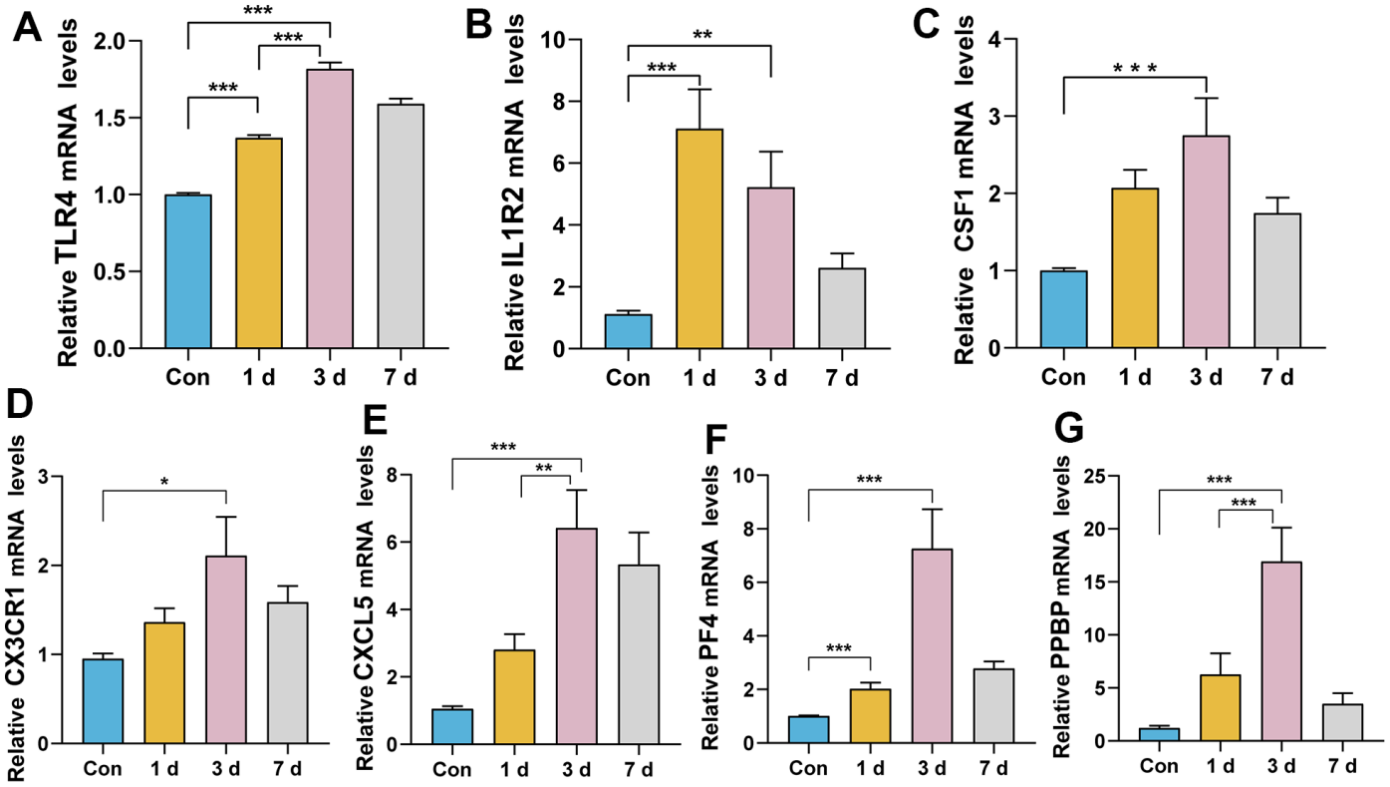
**Relative expression levels of the seven genes after ICH in mice.** (**A**) TLR4: ICH 3 d vs. Con, ICH 3 d vs. ICH1 d, ICH 1 d vs. Con, ***P< 0.0001. (**B**) IL1R2: ICH 3 d vs. Con, **P < 0.01; ICH 1 d vs. Con, ***P< 0.0001. (**C**) CSF1: ICH 3 d vs. Con, ***P< 0.001. (**D**) CX3CR1: ICH 3 d vs. Con, *P < 0.05. (**E**) CXCL5: ICH 3 d vs. Con, ***P< 0.0001; ICH 3 d vs. ICH1 d, **P < 0.01. (**F**) PF4: ICH 3 d vs. Con, ICH 1 d vs. Con, ***P< 0.0001. (**G**) PPBP: ICH 3 d vs. Con, ICH 3 d vs. ICH1 d, ***P< 0.001. (n = 6/group/time point).

RT-qPCR experiments were performed to investigate the expression levels of these genes after ICH in mice. The relative expression levels of *Pf4, Ppbp, Cx3cr1, Cxcl5, Csf1* and *Tlr4* were consistent with the transcriptome data. Compared to the control group and ICH 1d, all these genes were upregulated in ICH 3 d (Figure 6). The expression level of *Il1r2* peaked at ICH 1d and decreased in ICH 3 d, but was still higher than in control mice. This expression trend was most consistent with human ICH patients.

## DISCUSSION

Most of the findings on injury and repair after ICH has come from mouse models, but these mechanisms and therapeutic effects are difficult to verify in human patients. The most important result of this study is the identification of genes that are significantly regulated after ICH in both humans and mice: *Pf4, Ppbp, Cx3cr1, Cxcl5, Csf1, Il1r2*, and *Tlr4*. In addition, we predicted the potential regulatory networks with significant differences based on transcriptome and non-coding RNA (including miRNAs and circRNAs) sequencing before and after ICH injury in mice.

*Pf4*, *Ppbp*, *Cx3cr1*, *Csf1*, *Il1r2* and *Tlr4* are annotated as orthologs between human and mouse. Although *Cxcl5* (also known as *Scyb5*) is not annotated as an ortholog, its functions in humans and mice are very similar. These genes are therefore of great research value. As proteins, CSF1, CX3CR1, CXCL5, IL1R2, PF4 and PPBP were involved in cytokine-cytokine receptor interaction, while TLR4 was involved in the NOD-like and Toll-like pathways. They can easily be divided into two types: chemokines and inflammatory factors.

PF4 (also known as CXCL4), CXCL5, PPBP (also known as CXCL7) and CX3CR1 are chemokines. Depending on the structure, they belong to the CXC subfamily or the CX3C family. Chemokines are considered to be important cytokines in the central nervous system (CNS) and the immune system [14]. In the early stages of CNS inflammation, they can attract peripheral immune cells across the blood-brain barrier (BBB) to sites of inflammation and play a role in neuron-glial cell communication, synaptic transmission, neurogenesis and plasticity [15]. Research has demonstrated that chemokine aggregation indicates the presence of inflammation after ICH in both humans and mice [16]. Although the therapeutic strategy of neutrophil inhibition after ICH is promising, the specific chemokines and signaling pathways have not been elucidated [17].

Platelet factor 4 (PF4) is synthesized mainly in megakaryocytes and released in high concentrations when platelets are activated. It is involved in the promotion of thrombosis, atherosclerosis and anti-angiogenesis [18]. A previous study reported PF4 as a therapeutic target for atherosclerosis [19]. CXCL5 (chemokine (C-X-C motif) ligand 5) is synthesized and released by astrocytes and microglia in the brain [14], and the level of CXCL5 was found to increase significantly 5 hours after traumatic brain injury (TBI) [20]. CXCL5 promotes microglial activation and neutrophil infiltration after white matter injury in premature infants and it induces BBB and white matter lesions [21]. CXCL5 has also been identified as a potential serum biomarker for white matter lesions in preterm infants, glial hyperplasia in Alzheimer’s disease and multiple sclerosis (MS) [21–23]. Little is known about the function of CXCL5 after ICH, but its role is likely to be pro-inflammatory. Pro-platelet basic protein (PPBP) can be produced by platelets, monocytes and macrophages and is released at high levels by activated platelets [24, 25]. Under chronic stress conditions, the expression of vascular PPBP increases in mice and directs leukocytes to the injured vasculature [26]. Higher levels of PF4 and PPBP expression have also been observed in the blood of patients with depression [27]. Like PF4 and CXCL5, PPBP has received little research attention in the context of ICH, but is considered a potential risk factor for coronary heart disease in patients with hyperlipidemia. It is also a peripheral blood leukocyte biomarker associated with depression [26, 28]. C-X3-C motif chemokine receptor 1 (CX3CR1) is expressed on a variety of cells including microglia and neurons [29]. Unlike the relationships between other chemokines and receptors, the recognition between CX3CL1 and CX3CR1 is specific; their crosstalk is considered crucial in the communication between microglia and neurons, and they are involved in the control of neurogenesis, synaptic plasticity and cell proliferation [30]. Recently, CX3CR1 has been identified as a potential therapeutic target for hypertension and diseases related to autonomic nerve function [31].

Most cells of the innate and adaptive immune systems express proteins of the interleukin-1 receptor (ILR) and TLR families. ILRs can induce inflammation and promote the expression of immune-related TFs associated with the immune system. IL1R2 can inhibit IL-1β-induced neurotoxicity. Conversely, IL-1β can promote the release of IL1R2 [32]. In studies of ICH and ischemic stroke (IS), *Il1r2* has been implicated as an early response gene [5, 33]. Furthermore, IL1R2 is a diagnostic marker for acute myocardial infarction (AMI) and post-traumatic stress disorder (PTSD) [34, 35]. Moreover, TLR family proteins can also trigger inflammation. In the CNS, TLR4 is most abundant in microglia and functions as a mature membrane receptor. TLR4-mediated inflammation is one of the most important factors leading to brain damage [36, 37].

Colony stimulating factor 1 (CSF1) can promote the release of pro-inflammatory chemokines. It is an important regulator of macrophage homeostasis that can determine the functional features of cerebellar microglia. The functional regulation of microglial CSF1 is influenced by neurons [38]. Animals lacking CSF1 show loss of microglia and morphological changes in the cerebellum, as well as reduced motor learning and social capabilities [39]. In TBI mice, CSF1 has been considered as a therapeutic target over a long period of time (up to 3 months after injury). During this period, increasing CSF1 levels effectively improved cognitive function and long-term memory [40]. However, both TLR4 and CSF1R (the receptor for CSF1) can promote chemokine release [14, 25]. Based on the above analysis, we believe that the CSF1-centered ceRNA regulatory network after ICH has important implications for future research.

In conclusion, this study shows that the mouse model of ICH is the closest to human patients in terms of chemokines and inflammation. Further research in these two directions may be even more important. Moreover, the seven DEGs are likely to have additional functions worth exploring; for example, they may serve as early biomarkers to assess neurological outcomes after ICH. In addition, the regulatory network in which the DEGs are involved could be used as a therapeutic target after ICH to significantly ameliorate neural damage in mice and humans.

## CONCLUSIONS

This study provided a complete set of differentially expressed genes and regulatory networks in mice after ICH. We identified a valuable signaling pathway, circRNA-000895-miR-335-5p-*PPBP/CSF1/TLR4*; all the factors in this pathway are homologous in human and mouse, and the mRNAs involved were significantly up-regulated after ICH. Furthermore, we suggest that the study of the mechanisms of chemokines and inflammatory factors in animal experiments after ICH is of great relevance to humans. Further mechanistic studies based on this article may accelerate the progress of translational medicine research in the field of ICH.

### Limitation

This article detects differentially expressed mRNAs, circRNAs and miRNAs after ICH. Although the sample sizes of human and mouse were small, we were able to identify the DEGs common to both species after the disease. Further research is needed to develop a more detailed regulatory network of DEGs with translational significance after ICH. The expression levels of these genes in healthy individuals and those at high risk of ICH should be included to confirm whether they can be used as early biological warning signals; this will promote research into their regulation mechanism after ICH and determine their potential as drug targets for human non-infectious diseases.

## MATERIALS AND METHODS

### Animals

Eight-week-old male C57BL/6 mice weighing 20-25 g were purchased from Liaoning Changsheng Biotechnology Company (Liaoning, China). Mice were housed in the laboratory for at least 1 week. Mice food was purchased from the Animal Experimental Center of the First Affiliated Hospital of Harbin Medical University.

### Grouping and ICH models

Mice were randomly divided into two groups: Control group (Con, n=3) and ICH group (ICH, n=3). Collagenase was used to induce a murine model of ICH. The experimental method is described in a previous study by Wang et al. [41]. Briefly, anesthetized mice were fixed to a stereotaxic instrument and, after skin preparation, a longitudinal incision was made between the two ears. After positioning the skull with the stereotaxic instrument, a hole was drilled in the skull and 0.4μL collagenase was injected into the striatum with a micro-syringe. After waiting for absorption, the gap in the skull was filled with wax, the skull wiped with saline and the skin sutured. Successful establishment of the model was confirmed by the behavior of the mice; neurobehavioral injury was the inclusion criterion for the experiment.

### Sample preparation

Samples for gene expression profiling and mRNA, miRNA and circRNA analysis were prepared using the same treatment. C57BL/6 mice were deeply anesthetized with inhalation anesthetic machine using isoflurane at ICH 3 d. After the heart was exposed, the systemic circulation was perfused with saline. Brain tissue was removed and stored at -80° C.

### Messenger RNA isolation and microarray scanning

Brain tissue was pulverized with liquid nitrogen. TRIzol was used for mRNA extraction. RNA quantity and quality were measured, and RNA integrity was assessed by standard denaturing agarose gel electrophoresis. Total RNA from each sample was linearly amplified and labeled with Cyanine 3 (Cy3)-UTP. Subsequently, the labeled cRNA was then fragmented and hybridized, and 100 μL of hybridization solution was added to the gene expression microarray slide. The hybridized arrays were washed, fixed and scanned using a DNA microarray scanner (Agilent G2505C Scanner, Agilent Technologies, USA).

### MicroRNA isolation and microarray scanning

Sequencing was supported by Annoroad Gene Technology Co, Ltd. (China). The mirVana RNA Isolation Kit was used to isolate low-molecular-weight RNA from mouse brain tissue in ICH 3 d and control mice. The Illumina HiSeq 4000 platform was used to sequence miRNA expression profiles according to the manufacturer’s instructions.

### CircRNA isolation and scanning

After total RNA was extracted from mouse brain tissue, the purity and concentration of the total RNA samples were determined using the NanoDrop ND-1000 to confirm that the samples could be used in the next steps. Total RNA was digested with ribonuclease R (RNase R) (Epicentre Technologies, USA) to remove linear RNA and enrich circRNAs. The circRNAs were then amplified and transcribed into fluorescent cRNAs using a random priming method (Super RNA Labeling Kit, Arraystar, USA). The labeled cRNAs were purified using the RNeasy Mini Kit (Qiagen, USA). The concentrations and specific activities of the labeled cRNAs were measured using the NanoDrop ND-1000. Labeled cRNAs were fragmented and hybridized, and 50μL of hybridization solution was added to the circRNA expression microarray slide. The hybridized arrays were washed, fixed and scanned using the Agilent G2505C scanner, and the Agilent Feature Extraction software (version 11.0.1.1) was used to analyze the array images.

### RT-qPCR

After obtaining brain tissue from mice at different ICH time points (n=6), total RNA was extracted using a total RNA extraction kit (DP431, TIANGEN), and the concentration and purity were determined using a NanoVue Plus Micro-Volume UV-Vis spectrophotometer. The total RNA was then reverse transcribed into cDNA using a reverse transcription kit (FSQ-201, TOYOBO), and the concentration and purity of the cDNA were determined. Finally, 20μL of amplification reaction mix (QPS-201, TOYOBO) was prepared and the samples were introduced into an ABI StepOne Real-Time PCR system. The primer sequences are listed in Table 2. The ΔΔCT method was used to calculate the results. The data obtained were analyzed by one-way ANOVA. Tukey multiple comparison was used to test for statistical significance between groups.

**Table 2:** Primer sequences for genes differentially expressed in both humans and mice before and after ICH.

**Gene**	**Primer sequences (5’to 3’)**
TLR4	Forward: ATGGCATGGCTTACACCACC
	Reverse: GAGGCCAATTTTGTCTCCACA
IL1R2	Forward: TCTGGTACCTACATTTGCACAT
	Reverse: CTGTATCTTTCCATCAGCGTTG
CSF1	Forward: GGCTTGGCTTGGGATGATTCT
	Reverse: GAGGGTCTGGCAGGTACTC
CX3CR1	Forward: GAGTATGACGATTCTGCTGAGG
	Reverse: CAGACCGAACGTGAAGACGAG
CXCL5	Forward: TGCGTTGTGTTTGCTTAACCG
	Reverse: CTTCCACCGTAGGGCACTG
PF4	Forward: GGGATCCATCTTAAGCACATCAC
	Reverse: CCATTCTTCAGGGTGGCTATG
PPBP	Forward: ACGAATACCATCTCTGGAATCC
	Reverse: TTCTTCAGTGTGGCTATCACTT
GAPDH	Forward: AGGTCGGTGTGAACGGATTTG
	Reverse: TGTAGACCATGTAGTTGAGGTCA

### Statistical analysis

### Functional annotation of differentially expressed genes

The R package edgeR was used for differential expression analysis. Differentially expressed genes (DEGs) with |log2FC|>1 and adjusted p-values(p.adj)<0.05 were identified. Gene ontology (GO) annotation (BP: biological process, CC: cellular component, and MF: molecular function) and Kyoto Encyclopedia of Genes and Genomes (KEGG) enrichment analysis were used to select the major functional and biological pathways of DEGs with Benjamini-Hochberg (BH) p.adj <0.05. These genes were enriched using the R package clusterProfiler, and the R package GOplot was used to identify the top five BPs, CCs and MFs.

### Construction of regulatory network for differentially expressed miRNAs and genes

The edgeR package was used to identify differentially expressed miRNAs, and miRTarBase was used to find miRNA-gene interactions. A regulatory network of differentially expressed miRNAs and genes was then constructed. This network was visualized using Cytoscape software.

### Construction of regulatory network for differentially expressed circRNAs and miRNAs

The edgeR package was used to identify differentially expressed circRNAs, and miRanda and psRobot were used to estimate the miRNA binding sites of sheared circRNAs. The circRNA-miRNA regulatory network was then constructed using Cytoscape software.

### Bioinformatic analysis of a human disease model

Transcriptomes from human disease models were obtained from the Gene Expression Omnibus (GEO) database (GSE125512) and a volcano map was used to display DEGs with |log2FC|>0.5 and p.adj <0.05. Similarly, GO annotation and KEGG enrichment analysis were used to identify the primary functions and biological pathways of enriched DEGs with BH p.adj<0.05. The R package GOplot was used to highlight the five most significant KEGG pathways. In addition, miRTarBase was used to identify miRNA-gene pairs and the TRRUST (v2.0) database to identify transcription factor (TF)-gene pairs. A multifactorial regulatory network was then constructed.

### Integration of mouse and human regulatory networks

Homology analysis was used to identify a primary regulatory network containing seven genes, miRNAs and TFs in a combination of critical disease-associated functional molecules in mice and humans. KEGG enrichment analysis was then performed on the combined network. Finally, Cytoscape software was used to generate a functional network map based on homologous genes and key TFs.

## Supplementary Material

Supplementary Figures
