# Supplementary material for: Consistency of mouse models with human intracerebral hemorrhage: core targets and non-coding RNA regulatory axis
Source: Aging (Albany NY). 2024 Jan 24;16(2):1952–67. doi: 10.18632/aging.205473 (PMC10866413; doi:10.18632/aging.205473)
Supplement: Supplementary Figures [file aging-16-205473-s001.pdf]

SUPPLEMENTARY FIGURES

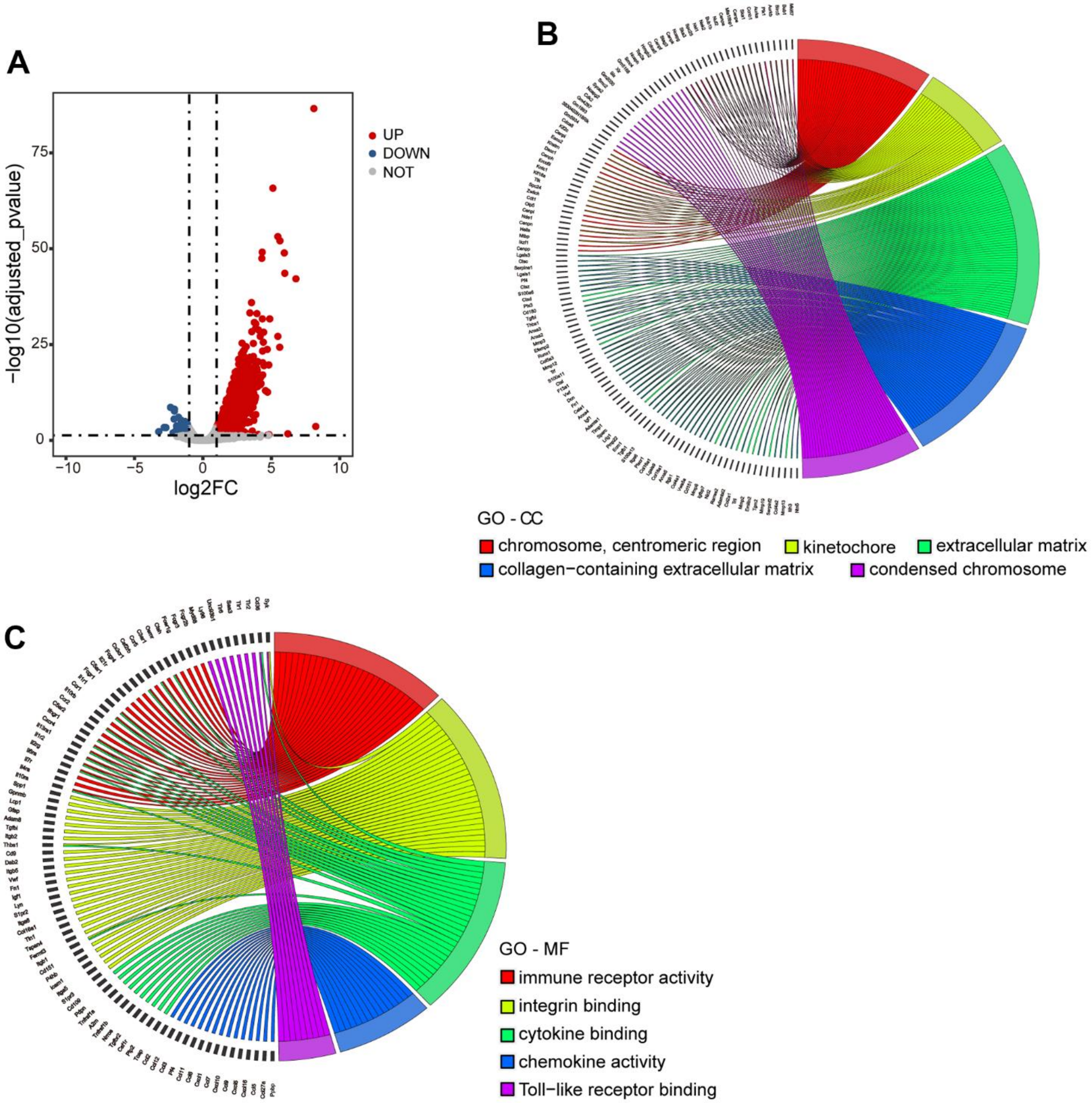

Supplementary Figure 1. Volcano mapping and other functional analyses of DEGs in ICH mice. (**A**) Volcano mapping of DEGs from ICH 3d compared to Con. (n=3, |log2FC|>1, p.adj<0.05). (**B**) Top 5 Cell Components (CCs) of GO and DEGs involved. (ICH 3d vs. Con, p.adj ≤2.21×10^{-11}). (**C**) Top 5 Molecular Functions (MFs) of GO and DEGs involved. (ICH 3d vs. Con,p.adj ≤2.95×10^{-7}).

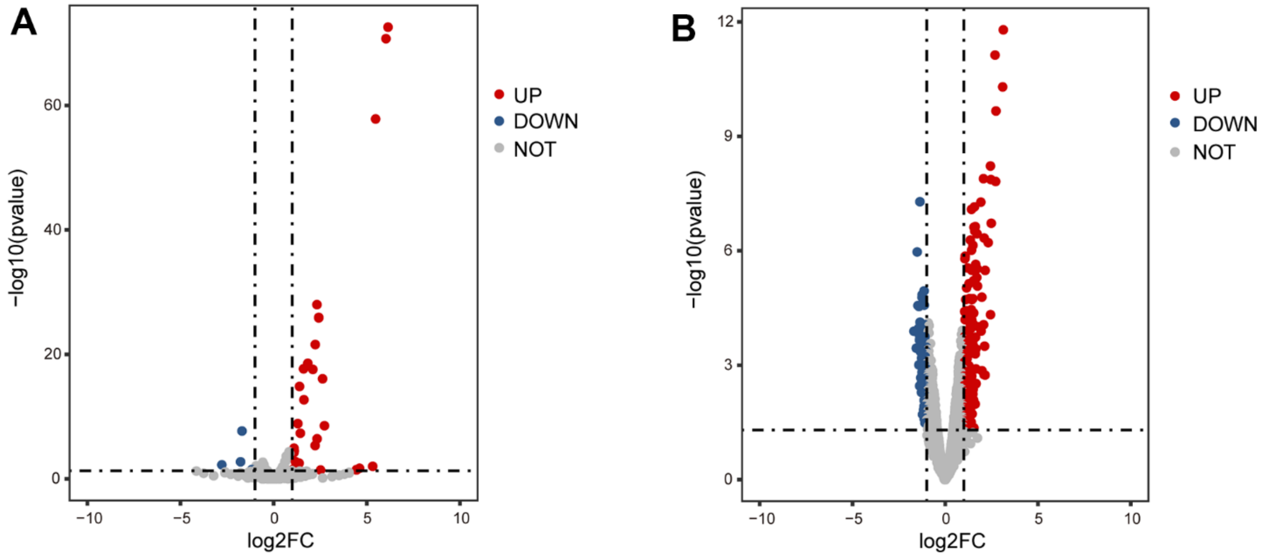

Supplementary Figure 2. Volcano mappings of miRNAs/circRNAs in ICH mice. (**A**) Volcano mapping of DE miRNAs from ICH 3d compared to Con. (n=3, |log2FC|>1, p <0.05). (**B**) Volcano mapping of DE circRNAs from ICH 3d compared to Con. (n=3, |log2FC|>1, p <0.05).

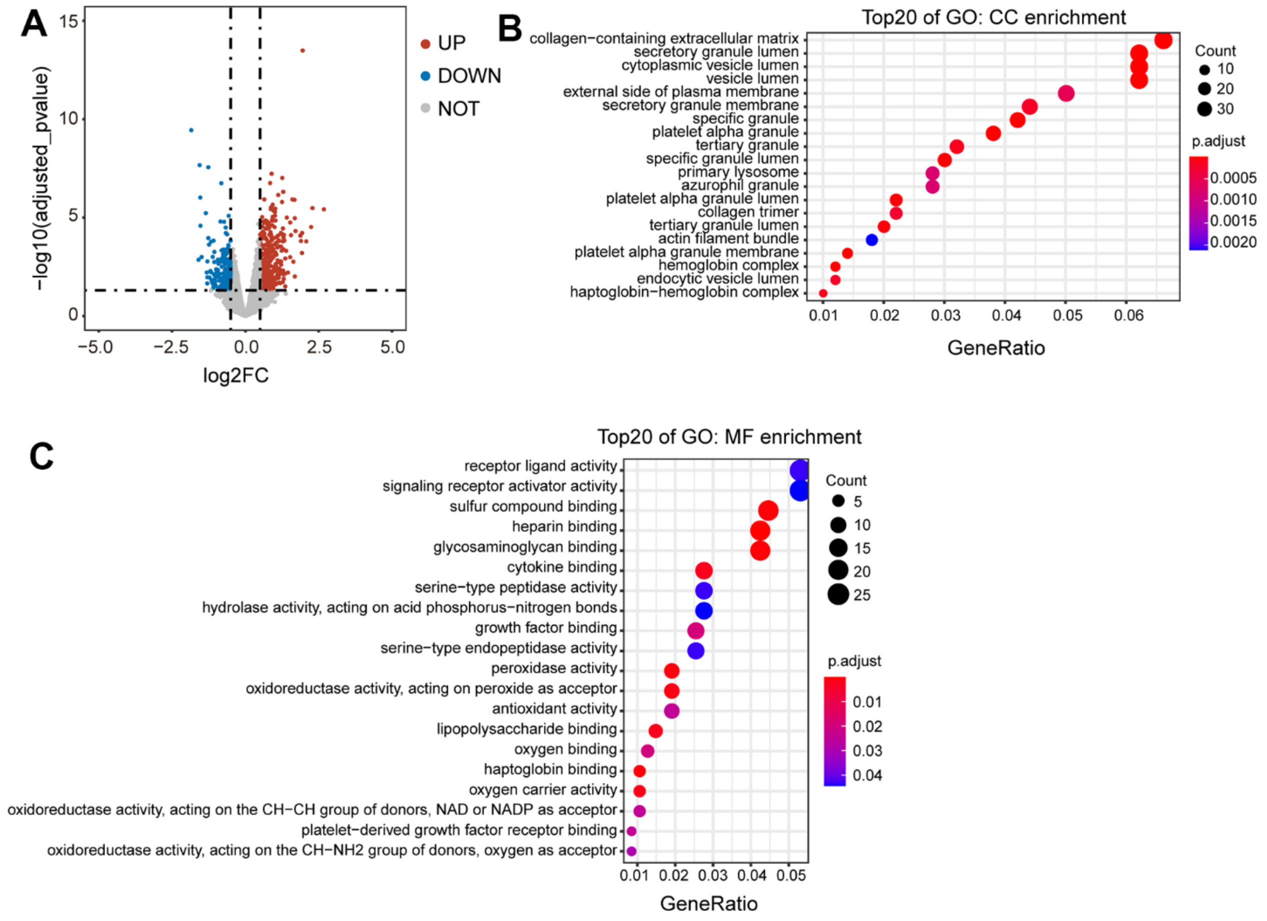

Supplementary Figure 3. Volcano mapping and other functional analyses of DEGs in ICH patients. (**A**) Volcano mapping of DEGs from ICH 72h compared to ICH 24h. (n=11, |log2FC|>0.5, p.adj<0.05). (**B**) Top 20 Cell Components (CCs) of GO and DEGs involved. (ICH 72h vs. ICH 24h, p.adj<0.001). (**C**) Top 20 Molecular Functions (MFs) of GO and DEGs involved. (ICH 72h vs. ICH 24h, p.adj ≤0.044).
